# Development of Cysteine-Free Fluorescent Proteins for the Oxidative Environment

**DOI:** 10.1371/journal.pone.0037551

**Published:** 2012-05-23

**Authors:** Takahisa Suzuki, Seisuke Arai, Mayumi Takeuchi, Chiye Sakurai, Hideaki Ebana, Tsunehito Higashi, Hitoshi Hashimoto, Kiyotaka Hatsuzawa, Ikuo Wada

**Affiliations:** Department of Cell Science, Institutes for Biomedical Sciences, Fukushima Medical University School of Medicine, Fukushima, Japan; The Beatson Institute for Cancer Research, United Kingdom

## Abstract

Molecular imaging employing fluorescent proteins has been widely used to highlight specific reactions or processes in various fields of the life sciences. Despite extensive improvements of the fluorescent tag, this technology is still limited in the study of molecular events in the extracellular milieu. This is partly due to the presence of cysteine in the fluorescent proteins. These proteins almost cotranslationally form disulfide bonded oligomers when expressed in the endoplasmic reticulum (ER). Although single molecule photobleaching analysis showed that these oligomers were not fluorescent, the fluorescent monomer form often showed aberrant behavior in folding and motion, particularly when fused to cysteine-containing cargo. Therefore we investigated whether it was possible to eliminate the cysteine without losing the brightness. By site-saturated mutagenesis, we found that the cysteine residues in fluorescent proteins could be replaced with specific alternatives while still retaining their brightness. cf(cysteine-free)SGFP2 showed significantly reduced restriction of free diffusion in the ER and marked improvement of maturation when fused to the prion protein. We further applied this approach to TagRFP family proteins and found a set of mutations that obtains the same level of brightness as the cysteine-containing proteins. The approach used in this study to generate new cysteine-free fluorescent tags should expand the application of molecular imaging to the extracellular milieu and facilitate its usage in medicine and biotechnology.

## Introduction

Various types of “tags” have been developed to analyze the specific event of molecules in focus. Particularly, the impact of fluorescent protein tags is enormous in biology in that they enable examination of the principle dynamics of molecules in living cells, and provide versatile tools to trace proteins at several levels including the whole body [1], [2], [3], [4], [5]. Also, this technology has allowed the identification of factors constituting complicated biological processes by genome-wide screening [6], [7] or even flow of information *per se* [8]. GFP isolated from *Aequorea victoria* was the first FP. Structural biology studies showed that the fluorescence was due to an autocatalytic cyclization of the GFP tripeptide S65-Y66-G67, which was embedded in an almost seamless ß-barrel structure, called β-can [9], [10]. Recently, the critical excited-state proton transfer was shown to associate with wagging of the phenol-ring of Y66 [11]. The original GFP was a dimer of low brightness and folded poorly at physiological temperatures in mammalian cells. However, after demonstration of GFP as a genetically encoded marker or tag in gene expression [12], [13], intensive efforts were undertaken to improve its brightness and to optimize its expression in mammalian cells [10]. The goal of these modifications was to render GFP inert. To this end, wild-type GFP has evolved into a monomeric, rapidly-folding stable protein under physiological conditions [14]. Genetic manipulations have also expanded the photophysical properties of GFP and created color variants as well as photokenetic variants [14], [15], [16], [17] such as photoactivatable (PA) GFP [15]. Meanwhile the identification of other sets of fluorescent proteins in different living species greatly expanded the possibilities of tagging technology [14].

Despite intensive improvement of FP techniques, their application to the milieu of the extracellular space has been limited. All FPs are cytosolic proteins but addition of a signal sequence caused them to cross the ER membrane, allowing expression of FP in the secretory pathway and analysis of the secretion process and products [18]. However, all fluorescent proteins except the DsRed family [19] possess multiple cysteine residues which can form disulfide bonds within the oxidative environment of the lumen of the ER, although the thiol groups of cysteine residues in GFP face the interior of the ß-can [20]. Mutation of the two cysteine residues to serines increased secretion efficiency [20], but the brightness of the mutant was greatly diminished. Recently, based on the rational that disulfide bonds would not be formed in the ER lumen if GFP folding precedes disulfide bond formation, an improved folding variant of EGFP [21], superfolder GFP [22], was shown to greatly reduce the formation of disulfide bonded oligomers even in the presence of the original two cysteine residues [23].

One might consider that the intact monomeric form of GFP is practically useful for fluorescent microscopic analysis since formation of disulfide bonds of a cysteine residue in a ß-can should destroy the tightly sealed structure and hence the covalent oligomers are not visible. This was the general assumption for usage of FP in the secretory pathway. However, this has not been rigorously tested. In this present study, we examined this and asked the question whether it was possible to eliminate all of the cysteine residues in fluorescent proteins by saturated mutagenesis without losing their brightness. We began with the brightest FP, SGFP2 [24], and the red fluorescent protein TagRFP [3] and successfully developed a set of fluorescent probes which included cfSGFP2 (a SGFP2 variant), and cfgTagRFP and cfgmKate2, which were derived from TagRFP. Their detailed analysis in living cells indicated that the presence of cysteine residues in the tag caused varying degrees of abnormalities in their diffusion and folding/targeting. These new tools should help in the study of the secretory pathway and this approach should also expand the possibilities of molecular imaging.

## Results

### cfSGFP2, a Cysteine-free GFP Variant

**Figure 1:**
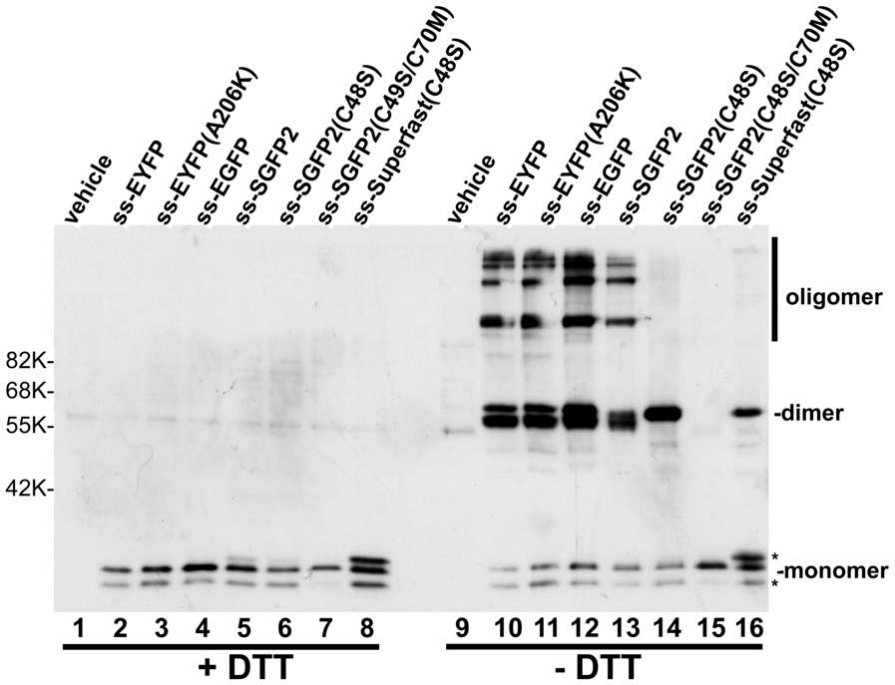
Oligomerization of GFP family proteins in the ER. Various GFP family proteins as indicated in the figure were targeted to the ER by a signal sequence (“ss”) and subjected to SDS-PAGE after SDS treatment at 70°C for 10 min with (lanes 1–8) or without (lanes 9–16) reduction as described in Materials and Methods. Shown is an immunoblot probed with anti-GFP antibody. The position of each oligomer is indicated. Vehicle: mock-transfection. *: unidentified bands. The numbers to the left indicate the positions of molecular mass markers. When molecules contained two cysteine residues, they formed higher molecular mass oligomers (lanes 10, 11, 12 and 13) whereas molecules containing a single cysteine formed a dimer (lanes 14 and 16). The monomer form SGFP2(C48S/C70M) (lane 15) is named cfSGFP2.

When GFP variants such as EGFP or its color variants were expressed in the ER by the addition of the signal sequence (ss) of α1-antitrypsin and the cells then incubated in the presence of brefeldin A to inhibit ER exit, most of the variants formed covalently-linked oligomers (Figure 1, lanes 10 and 12). Even a “monomeric mutation”, A206K [25], that greatly reduces subunit association by introducing charge-repulsion at the dimer interface had no effect on oligomerization (lane11).

**Figure 2:**
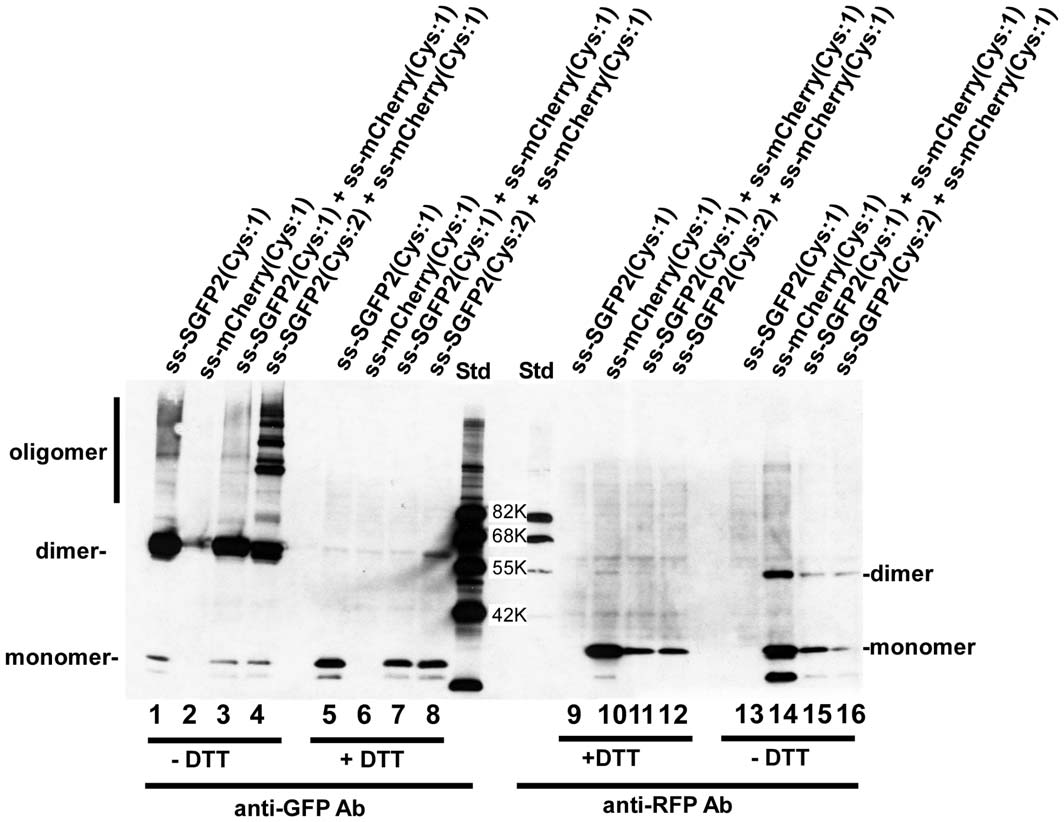
Oligomer formation of fluorescent proteins is specific. The indicated fluorescent proteins were expressed in cells and the cells were then lysed and analyzed by immunoblotting using either anti-GFP antibody (lanes 1–8) or anti-RFP antibody (lanes 9–16). As in Figure 1, samples were SDS-treated with (lanes 5–12) or without (lanes 1–4 and 13–16) reduction. “Cys:1″ indicates fluorescent proteins containing C48S (SGFP2) or S3C (mCherry) and SGFP2“Cys:2″ is the wild type SGFP2. In the lanes where two proteins are listed, both expression plasmids were cotransfected. Std: molecular mass markers as indicated in the figure. As neither antibody cross-reacted and indeed showed no reaction in lane 6, then the band in lane 2 is assumed to be a leaked sample from the adjacent lanes. mCherry(Cys:1) formed a disulfide bonded dimer (lane14). Since the molecular masses of SGFP2 and the mCherry oligomer are different (lane 1 or 5 versus lane 10 or 14), then heterooligomers consisting of SGFP2 and mCherry should be distinguishable from each homooligomer. As the band profiles of lane 1 (SGFP2(Cys:1) alone) and lane 3 (SGFP2(Cys:1)+mCherry(Cys:1)) were almost identical, then a disulfide bond was not formed between SGFP2(Cys:1) and mCherry(Cys:1). The same is true for lanes 14 (mCherry(Cys:1)) and 15 (SGFP2(Cys:1)+mCherry(Cys:1)) and lane 16 (SGFP2(Cys:2)+mCherry(Cys:1)).

To understand whether this oligomerization was caused by the intrinsic intersubunit affinity of GFP or occurred stochastically due to the high redox-potential of the ER lumen, we examined whether GFP formed a mixed dimer with another thiol-containing protein (Figure 2). mCherry is a red-fluorescent protein with no cysteines. When we added a cysteine residue at the N-terminus of mCherry, the mutant (ss-mCherry(Cys:1)) also formed a dimer (Figure 2, lane14). Similarly, a mutant SGFP2 (ss-SGFP2(C48S)) that contains C70 but not C48, formed a covalent dimer (Figure 1, lane 14). Co-expression of both single cysteine mutants showed no mixed dimer consisting of SGFP2 and mCherry (Figure 2, lanes 7 and 15). This indicated that oligomerization of SGFP2 was caused by its intrinsic aggregation property, which is almost eliminated in the folded form [25]. Consistent with this, the superfast mutation (F64L/N105Y/E124V/Y145F) [26] reduced the extent of dimer formation (Figure 1, lane16). Superfast GFP folds about 1.8-fold faster than superfolder GFP in vitro [26].

We started our mutations with SGFP2, one of the brightest GFP variants [24], and performed site-saturated mutagenesis on C48 and C70 based on the assumption that some amino acids would mimic the role of cysteine in excitation-induced photon emission. Serine residues have been commonly used to replace cysteines because of their chemical similarity and indeed C48, which resides outside the β-can structure, could be replaced with serine without loss of brightness. In the case of C70, site-saturated mutagenesis showed that methionine was the only amino acid that could functionally substitute C70, whose thiol group is located inside the β-can. The SGFP2 variant possessing the double mutant, C48S/C70M, was called cf(cysteine-free)SGFP2 (Figure 1, lane 15).

**Figure 3:**
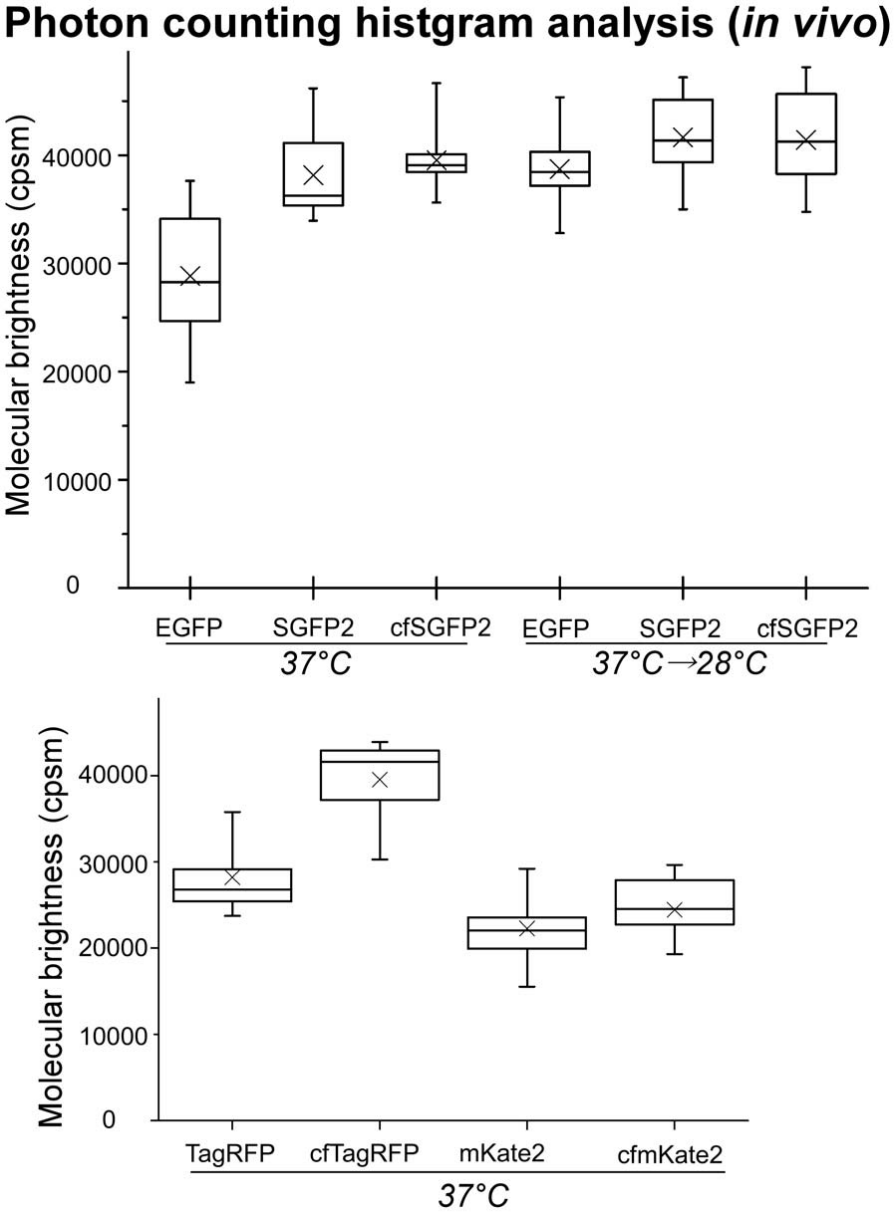
Molecular brightness of cysteine-free fluorescent variants. Brightness per molecule was estimated in the cytosol of living cells as described in Materials and Methods, and plotted as box charts. Each fluorescent protein was expressed in a chamber stage for 3 hr at 37°C or for 3 hr at 28°C after the 3 hr expression period at 37°C. Twenty measurements were carried out for each protein. A box chart shows mean (cross), median (horizontal line), 25–75 (box) and 95 percentiles (bars) of molecular brightness.

In contrast to the extremely low brightness of the C48S/C70S GFP mutant [20], cfSGFP2 apparently retained most of its brightness. To quantify the specific brightness, we analyzed the photon counting histogram (PCH). This technique was developed to account for the fluctuations in fluorescence amplitude for molecules diffusing through an observation volume [27]. Here, we used a global PCH analysis protocol that simultaneously calculates time-dependent decay of correlation function for accurate time-indepepdent estimation of molecular brightness [28]. We beads-loaded the expression plasmids for EGFP, SGFP2 and cfSGFP2 in COS7 cells and incubated for 3 hr at 37°C, then recorded intensity fluctuation data consisting of 1–4×10^6^ photons from single measurements. As shown in the upper panel of Figure 3, cfSGFP2 showed comparable in vivo brightness as SGFP2. Brightness of EGFP (average 28831 cpsm) was lower than that of SGFP2 (38178 cpsm) or cfSGFP2 (39524 cpsm) in the measurements. However, further incubation at 28°C increased the brightness of EGFP (38727 cpsm) to the same level as SGFP2/cfSGFP2, suggesting that the improved maturation property of SGFP2 was retained in cfSGFP2. In addition, one of the superior properties of SGFP2 is its photostability [24]. This stability of SGFP2 (1.7-fold compared to EGFP) was also not impaired in cfSGFP2 (2.0-fold) (Table 1).

**Table 1:** Photo-chemical properties of fluorescent proteins.

	Excitation/emission maxima (nm)	QY^a^ (nm)	ε^b^(mM^−1^cm^−1^)	Brightness		pKa	τ _leach_ c (*in vivo*)
				QY_×_ε	*in vivo* d		
EGFP	489/510	0.67 (460)	55	37	28831	5.4	1
SGFP2	497/517	0.77 (460)	40	31	38178	5.4	1.7
cfSGFP2	493/517	0.75 (460)	45	34	39524	5.4	2.0
TagRFP	556/585	0.51 (520)	90	46	28198	3.1	1
cgfTagRFP	555/584	0.42 (520)	86	36	39532	2.9	0.9
mKate2	586/633^e^	0.40^e^	63^e^	25^e^	22297	5.4^e^	1.7
cgfmKate2	584/628	0.47 (530)	55	26	24441	4.6	1.4

a Absolute quantum yield corrected with reabsorption and reemission (the excitation maximum in parentheses).

b Extinction coefficient measured with the absorbance maximum.

c Photostability. Time to bleach 1/e of fluorescence relative to EGFP (SGFP2, cfSGFP2) or TagRFP expressed in COS7 cells (cgfTagRFP, mKate2 and cgfmKate2).

d Photon counts per second per molecule (cpsm) of fluorescent proteins in COS7 cells at 37°C (Figure 3).

e Cited literature ([41]).

### Cysteine Residues in SGFP2 Restrict Free Diffusion

**Figure 4:**
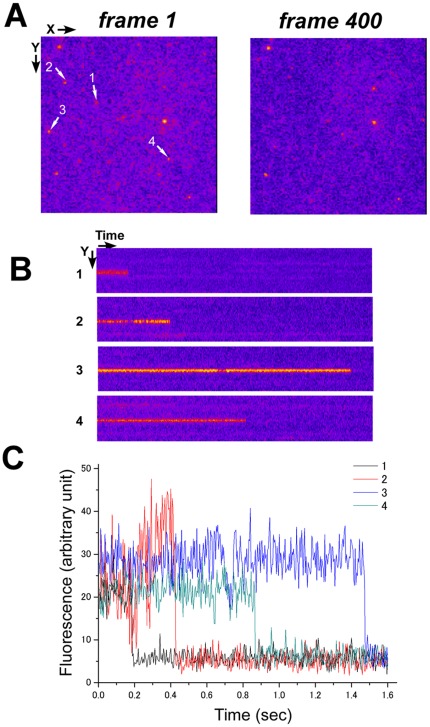
Single step photobleaching of SGFP2 in the ER. Cells expressing ss-SGFP2 were extensively fixed for 4 days with paraformaldehyde as described in Materials and Methods and single molecules were directly visualized by total reflection fluorescence microscopy. (A) When 400 frames were recorded at a frame rate of 250 Hz, 4 spots disappeared from the first frame in the recorded 1.6 sec (indicated by arrows). The width and height of an image is 13.6 µm. (B) Kymograph of each spot (1–4) from panel A. A single pixel image of the Y axis is lined along progression of time. (C) Quantification of the fluorescent signal in panel B. All spots were photobleached in a single step.

Since the core β-can structure of GFP should be severely damaged by disulfide bond formation, it is unlikely that the disulfide bonded GFP is fluorescent. We tested this by measuring the photobleaching property of SGFP2 at a single molecular level (Figure 4). If disulfide-bonded oligomers are fluorescent, photobleaching of the oligomers should occur in a stepwise fashion. However, when we measured the photobleaching kinetics from four hundred streaming images of a single SGFP2 molecule tightly immobilized in the ER under evanescence illumination, at least four molecules disappeared (Figure 4A). A kymograph of each spot (Panel B) and its quantification (Panel C) showed that there was no stepwise photobleaching.

**Figure 5:**
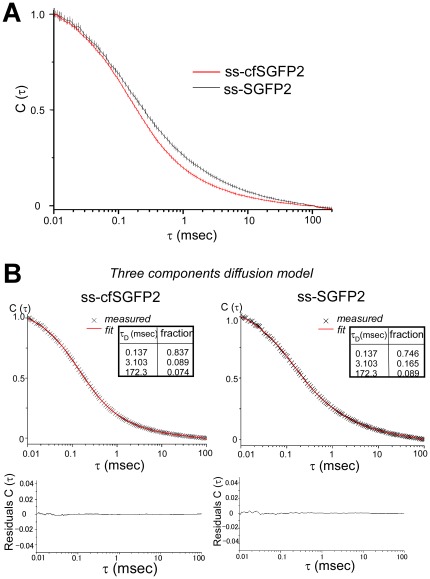
Cysteine restricts simple diffusion in the ER. The autocorrelation function of SGFP (n = 146) or cfSGFP2 (n = 172) in the ER of living cells was measured as described in Materials and Methods. Shown are the normalized mean (solid lines) and CI95 (perpendicular bars) of the measurements (A). Best fits and residuals for diffusion time *τ_D_* and fraction were obtained when the three component diffusion model was applied (B). ss-cfSGFP2 showed a steeper decay compared to ss-SGFP2.

This data indicated that fluorescent GFP molecules should all be monomers, suggesting that fluorescent molecules of SGFP2 and cfSGFP2 should behave identically. However, when we compared the autocorrelation function of SGFP2 and cfSGFP2 in the ER, we found a large difference in the decay profile of FCS (fluorescence correlation spectroscopy) (Figure 5A). Decay of autocorrelation was significantly slower in SGFP2 compared to cfSGFP2. To analyze divergence from normal diffusion, we considered three diffusional models. First, “anomalous exponents” was introduced for fitting [4], [29], [30]. In nonhomogeneous media, mean square displacement *<*Δ*r^2^>* of a particle does not linearly expand with time *t* very often but rather, their relationship could be assumed to exhibit power law scaling with
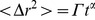
(1)where *Γ* is a factor usually referred to as the transport (diffusion) coefficient. In this equation, the magnitude of aberrant motion appears as an exponent *α* (*α*<1). When we applied this model, SGFP2 (Figure S1A, *α* = 0.667) exhibited more divergence from normal diffusion than cfSGFP2 (*α* = 0.792). However, as profiles of residuals showed some forms of systematic error, this model appears to be inappropriate.

It is also possible to take into consideration the realistic geometry of the ER as a FCS model. Autocorrelation function *G(τ)* in the space can be decomposed into a product of each autocorrelation function *g_x_(τ)*, *g_y_(τ)* and *g_z_(τ)* in axial dimension,
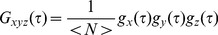
(2)


If motion of the cargo is limited by membranes in an optical confocal volume, *g_i_(τ)* is affected by the confined parameter, *d_i_/r_i_*, where *d_i_* is the confined volume diameter and *r_i_* the 1/*e^2^*-radius of the detection volume in *i*-axis. *G_xyz_(τ)* can be approximated as equations (3–6) of *Methods* for *d_i_/r_i_* ≤8 [31]. The optical geometry should be independent of the cargo property; however, *d_i_* becomes shorter if luminal volume is narrowed by accumulation of nonfluorescent cargo. When we made this model fitted to the curve, the confinement parameter, *d_y_/r_xy_* of ss-SGFP2 was 23% smaller than ss-cfSGFP2 (Figure S1B). However, the larger divergences from the best fits also indicate that this model does not seem to explain the feature of the cargo motion.

Aberrant decay profiles of these ER cargo molecules were best resolved by the three-component diffusion model (Figure 5B) as previously used [32]. The best-fit of ss-SGFP2 indicated one-fourth of molecules showed aberrantly slow motion. The fraction of simple diffusion (*τ* = 0.137 msec) was improved up to 84% in cfSGFP2 (left panel). Compared to the previous two models, divergence from the best-fits was very small and appears to be stochastic. The results clearly indicate that cysteine in the fluorescent protein contributes to the generation of slowly diffusing populations.

In contrast, most of the thiols are intact in the cytoplasm due to the low redox potential. However, there are classes of enzymes which react directly with thiols, such as thioredoxin [33]. They regulate various biological processes by catalyzing disulfide bonds [34]. We thus considered the possibility that perhaps the cysteine residues of GFP may disturb normal diffusion in the cytoplasm. When we compared normalized autocorrelation curves (Figure S2A), decay profiles containing a time domain (centered at 0.3–0.4 msec) showed a small but statistically significant difference (Panels A and B). This would not be due to the outlier since the difference was observed both in mean and median (Panel A). Fitting revealed that elimination of cysteine significantly reduced the aberrantly diffusing populations (Panel C).

### Effect of Cysteine Residues in a Tag on Folding and Targeting of a Fusion Protein

**Figure 6:**
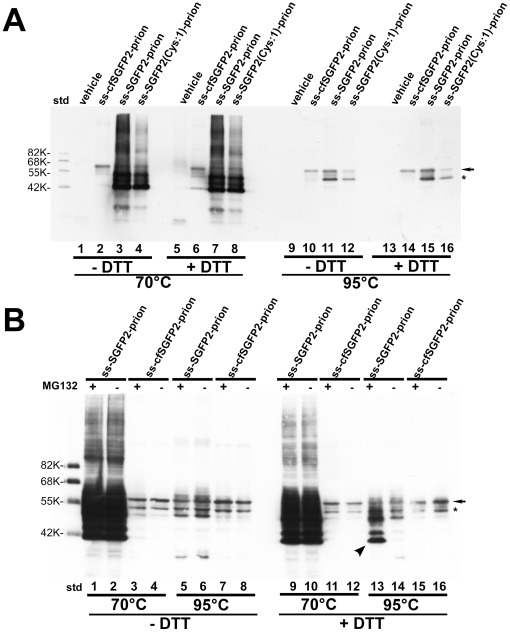
Formation of an aberrant SDS-resistant structure in SGFP2-prion fusion protein but not in cfSGFP2-prion fusion protein. Lysates of cells expressing the prion fusion proteins indicated in the figure were analyzed by Western blotting as described in Materials and Methods. (A) SDS-solubilized lysates were treated either without DTT (lanes1–4 and 9–12) or with DTT (lanes 5–8 and 13–16) at either 70°C or 95°C for 10 min. Prion protein fused to ss-cfSGFP2 migrated on the gel at the expected size of 58 kDa under all conditions (lanes 2, 6, 10 and 14). In contrast, prion protein fused to ss-SGFP2 or ss-SGFP2(C48S) showed intense fast migrating bands (lanes 3, 4, 7 and 8) when samples were treated at 70°C, but not at 95°C (lanes 11, 12, 15 and 16). * indicates an unknown form of the prion fusion protein which was not consistently observed. (B) Cells were incubated with or without 100 nM MG132 for 4 hr as indicated in the figure and, as in (A), cell lysates were treated under four different conditions. When MG132 was included, a set of faster migrating bands was not diminished completely by a 95°C treatment (lane13, *arrowhead*).

When a protein tag is genetically fused to a protein of interest, the tag should not disturb the structure of the fused protein. However, in some cases, tagging could severely interfere with the proper folding of the protein. When prion, a GPI-anchored protein, was fused to ss-SGFP2, we obtained an unusual SDS-PAGE pattern in either non-reducing or reducing gels (Figure 6A, lanes 3 and 7). In this case, there was no obvious large oligomer in non-reducing SDS-PAGE (-DDT). Rather, the major band migrated much faster than the expected size and showed an apparent mass of ∼42 kDa. In this construct, a fluorescent protein containing the cleavable signal sequence of α1-antitrypsin was joined to the N-terminus of the mature prion protein at R25 since prion contains a GPI-anchoring domain at its C-terminus and a cleavable signal sequence.

In contrast, as shown in lane 2 of Figure 6A, the ss-cfSGFP2-fused prion protein migrated normally on a gel at its expected size of 58 kDa. We thought that the aberrant pattern of ss-SGFP2-prion may have been caused by formation of a SDS-resistance compact structure in the fusion protein. As it is known that some membrane proteins can only be resolved by SDS-PAGE if heated at a higher temperature [35], we increased the temperature for the SDS treatment. As shown in lanes 11 and 15, 95°C-treatment almost completely changed the aberrant pattern observed in lane 3 or 7, and produced a 58 kDa (*arrow*) and a 45 kDa band (*). The size of the larger band was identical to the mass of the ss-cfSGFP2-prion fusion protein and the smaller band was also visible in lane 10. A mutant SGFP2(C48S) did not improve the aberrant pattern although the extent of higher molecular weight oligomers were reduced (lanes 4 and 8). This result strongly indicates that even a single cysteine residue in SGFP2 could interact with the prion polypeptide during folding and form an unusually stable compact structure.

We speculated that perhaps misfolded forms of the ss-cfSGFP2-prion fusion proteins may be disposed by the quality control mechanism of the ER. To this end, we inhibited proteasomal degradation using the tri-leucine derivative, MG132 [36] and examined if misfolded forms of the fusion proteins accumulated. However, no apparent change was observed in the ss-cfSGFP2-prion fusion protein (Figure 6B, lanes 3 and 11). In contrast, for the ss-SGFP2-prion fusion protein the same set of faster migrating bands as observed by 70°C SDS treatment was observed by a 95°C treatment in the MG132-treated cells (*arrowhead*, lane13). Since they were not present in the absence of MG132 treatment (lane 14), then these must be disulfide-bonded misfolded forms that are highly resistant to SDS and usually disposed by ER-associated degradation.

**Figure 7:**
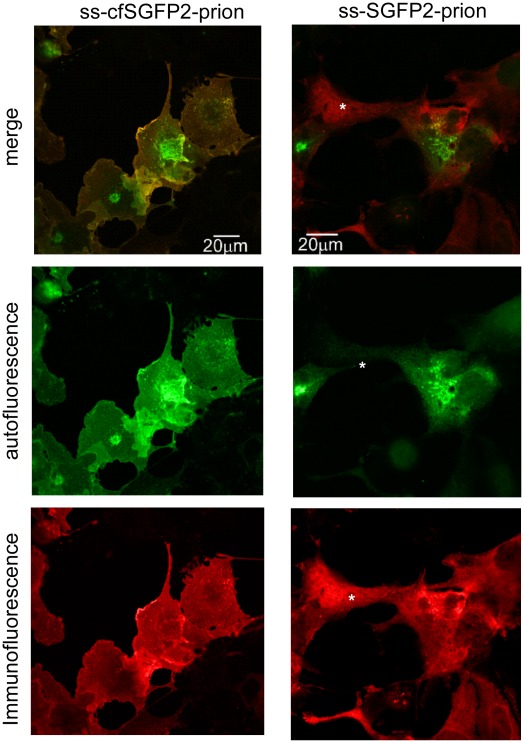
Mistargeting of SGFP2-tagged prion. COS7 cells expressing prion protein fused to either ss-cfSGFP2 (*left*) or ss-SGFP2 (*right*) were immunostained with GFP antibody and the epifluorescent (*red*) or autofluorescent (*green*) images were observed by confocal microscopy as described in Materials and Methods. In ss-cfSGFP2-prion fusion protein, most of the GFP signal showed colocalization with the anti-GFP antibody signal (*merge, left*) with the exception of a perinuclear, Golgi-like region (see *Results*). In contrast, the ss-SGFP2-prion fusion protein showed markedly distinct images (*merge, right*). The confocal pinhole was set to 1.0 Airy Units.

When the cellular localizations of ss-cfSGFP2-prion protein and ss-SGFP2-prion protein were compared in COS7 cells, marked differences were observed (Figure 7). In the case of the ss-cfSGFP2-prion fusion, the fluorescence signal was clearly observed at the plasma membrane in addition to a concentrated region at the Golgi-like structure and the overall pattern (*middle*) was very similar to the immunofluorescence pattern obtained by incubation with anti-GFP antibody (*bottom*). The only difference was in the region of the Golgi where autofluorescence was intense but lacked the GFP antibody signal. This could be explained by steric hindrance of the ss-cfSGFP2-prion fusion protein due to its extensive concentration at the Golgi region such that the antibody had no access to the fusion protein. In contrast, cell surface labeling of prion protein fused to ss-SGFP2 was very poor and the cell surface image was barely observed (right panels). In this case, fluorescence was inconsistent and in some cells (*asterisk*) only a very faint autofluorescence was observed. Most of the signal appeared to be in the Golgi and ER-like reticular structures. When COS7 cells expressing the ss-cfSGFP2-prion fusion protein were briefly exposed to Cu^2+^ at pH 5 to induce cytotoxic damage [37], large aggregates were formed on the surface of cells, leading to apoptosis of some cells (Supplementary movie).

### Cysteine-free TagRFP Variants cgfTagRFP and cgfmKate2

**Figure 8:**
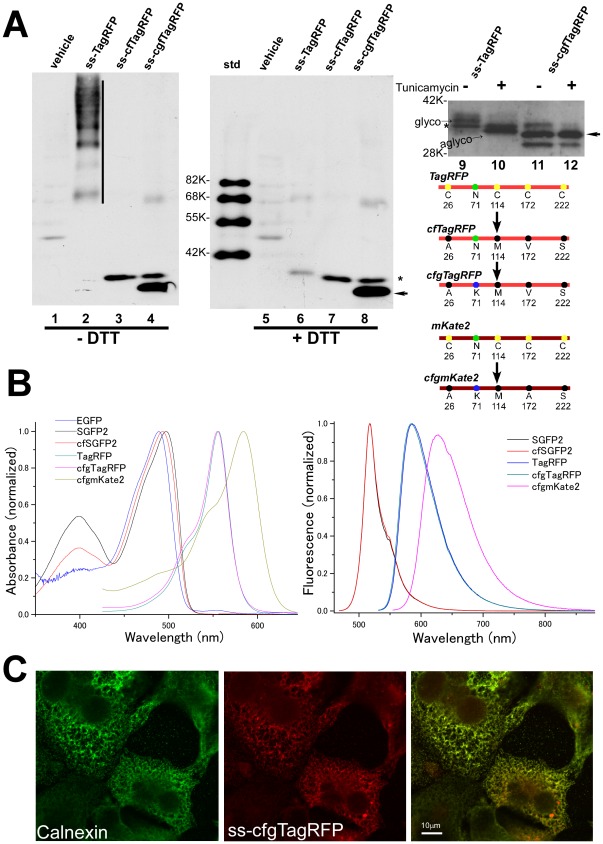
Development of cgfTagRFP and cgfmKate2. Lysates of cells expressing ss-TagRFP, ss-cfTagRFP and ss-cgfTagRFP were subjected to immunoblot analysis using anti-TagRFP antibody (A). As with the GFP family proteins, ss-TagRFP showed extensive oligomers under non-reducing conditions (lane 2, *perpendicular line*). This is in contrast to ss-cfTagRFP (lanes 3 and 7) or ss-cgfTagRFP (lane 4 and 8) which showed identical patterns. The major band of ss-cgfTagRFP (*arrow*) migrated faster than ss-cfTagRFP. Tunicamycin treatment (5 µg/mL) for 8 hr caused faster migration (lane 10, “*aglyco*”) of ss-TagRFP (lane9, “*glycol*”). In contrast, ss-cgfTagRFP showed no difference in mobility (lanes 11 and 12). Bands of unknown origin that are inconsistently observed with ss-cgfTagRFP (lane 8) or ss-TagRFP (lanes 9 and 10) are indicated by an asterisk (*). Schematic drawings of mutations in cgfTagRFP are also shown. (B) Excitation and emission spectra of newly developed fluorescent proteins. Normalized absorption (*left*) or emission spectra (*right*) of the purified fluorescent proteins. (C) Localization of ss-cgfTagRFP in cells treated with brefeldin A. Fluorescence of ss-cgfTagRFP almost completely matched with anti-calnexin antibody immunofluorescence.

TagRFP [3] and mCherry [38] are two of the brightest, most frequently used red fluorescent proteins. While TagRFP has superior properties compared to mCherry in that TagRFP has a higher quantum yield and is less prone to aggregate, TagRFP contains four cysteine residues which are also conserved in its color variants, TagBFP [39], TagGFP and mKate/mKate2 [39], [40]. Similar to the GFP family of proteins, TagRFP also formed large covalent oligomers in the ER (Figure 8A, lane2). No monomer band was observed for TagRFP and the degree of oligomerization was more extensive for TagRFP than that seen for GFP and is most probably due to the fact that TagRFP contains four cysteine residues. To eliminate the cysteine residues from TagRFP, serine substitution of the C222 that is located outside the β-can structure was first applied to TagRFP and it was confirmed that this mutation had no effect on brightness. We then performed several rounds of saturated mutagenesis to look for replacement of the cysteine residues of TagRFP(C222S). We found that one set of mutations, C26A/C114M/C172V/C222S, could retain enough brightness and we called this TagRFP mutant cfTagRFP (Figure 8A, lane3).

Since cfTagRFP contains a single N-glycosylation site at N71, we wondered if this amino acid could be replaced with another amino acid to generate a second fluorescent mutant of TagRFP. To this end, we performed a saturated mutagenesis on N71 and found that replacement of N71 with lysine generated another mutant which retained a brightness almost equal to cfTagRFP and we named it cgf(cysteine- and glycan-free)TagRFP. The difference in the migration of the major bands in lane 7 and 8 of Figure 8A would reflect the mass of a single N-linked oligosaccharide. To confirm this, we used tunicamycin, which inhibits N-glycosylation by blocking synthesis of dolichol-core oligosaccharides, to treat the cells. ss-TagRFP synthesized in the treated cells showed a small but clear shift (“aglyco”, lane 10) whereas ss-cgfTagRFP showed no change in mobility (lane 12), confirming that the N71 of TagRFP was actually N-glycosylated. Specific brightness analysis showed that cgfTagRFP emits comparable amount of photons as TagRFP upon excitation (lower panel of Figure 3). The emission spectrum of cgfTagRFP was undistinguishable to TagRFP (Figure 8B). Transfection of a plasmid encoding ss-cgfTagRFP in which a signal sequence was added to cgfTagRFP clearly highlighted the ER as did calnexin when export from the ER was inhibited by brefeldin A (Figure 8C).

mKate2, a near-infrared variant of TagRFP, has been reported as a useful tool for whole body imaging [41]. We confirmed that the N71K mutation used in cgfTagRFP was also effective in retaining the fluorescence of mKate2, but the same set of cysteine replacements as was done to generate cgfTagRFP abolished the brightness of mKate2 (our unpublished observation). By examining the effects of individual cysteine substitutions, we observed that C172V failed to retain the brightness of mKate2. Hence we performed another round of site-saturated mutagenesis where C172 was replaced with an alanine residue. This mKate2 variant C26A/C114M/C172A/C222S was called cgfmKate2. PCH analysis of cgfmKate2 showed that the in vivo brightness was unchanged from mKate2 (Figure 3).

### Photochemical Properties

To examine photochemical properties of a set of cysteine-free fluorescent proteins as described above, we carried out several measurements of the purified proteins. cfSGFP2 showed brightness very similar to SGFP2 with a higher quantum yield (Table 1). As with SGFP, cfSGFP2 showed red-shift absorbance compared to EGFP, albeit the shift of cfSGFP2 was slightly smaller than SGFP (Figure 8B anf Table 1). It should be noted that a characteristic peak of SGFP2 at 396 nm, which is not prominent in EGFP, was reduced by 22% in cfSGFP2.

In vivo molecular brightness of cgfTagRFP was comparable to TagRFP (Figure 3 and Table 1) and the isolated protein also showed similar brightness (78%) compared to TagRFP (Table 1). The bleaching time constant of cgfTagRFP was only slightly lower than TagRFP. pKa values of both red fluorescent proteins were near 3 (Figure S3 and Table 1), indicating that the acid-resistance of the fluorophore was not lost by the elimination of cysteine. This property was not conserved in the red-shift variant, mKate2 whose pKa was 5.4 [41]. In cgfmKate2, the pKa was reduced to 4.6 (Figure S3 and Table 1). We did not include the results of mKate2 in Table 1 since our mKate2 preparations were unstable when isolated from bacteria. Consistent wth the comparable in vivo molecular brightness of cgfmKate2 to the parent protein, the quantum yield of cgfmKate2 (0.47) was comparable to the reported quantum yield (0.40) of mKate2.

## Discussion

GFP technology has become a routine basic methodology in various fields of the life sciences. This has enabled analysis of the dynamics of molecules in the living state. For example, diffusion is the most basic parameter of any reaction and only the development of GFP revealed how genetically coded molecules actually propagate in cells. However, usage of the GFP tag in the secretory pathway seems to be limited. We considered that the limitation of GFP technology in the secretory pathway was related to the anomalous property of motion in the lumen of the ER and the reactivity of the cysteine residues in GFP family proteins in the oxidative environment. To overcome these problems, in this present study we have developed cysteine-free fluorescent tags and identified a set of cysteine replacement mutants that could still retain the brightness of their wild type counterparts. We have shown that the GFP mutant cfSGFP2 dramatically improved the diffusional property and inertness as a tag. To expand the repertoire of fluorescent tags that would be useful for analysis of the secretory pathway or extracellular space, we further generated cgfTagRFP and cgfmKate2 as cysteine-free (cf) variants of TagRFP and mKate2, respectively.

In early studies aimed at improving fluorescent proteins as tags, efforts were focused on eliminating the intrinsic oligomerization property of GFP or RFP (DsRed or eqFP578) family proteins [3], [25], [42]. These strategies were essentially based on creation of charge repulsion [42] and/or elimination of hydrophobic residues at subunit interfaces [25]. As described previously [23], covalent oligomers in the ER are apparently formed before obtaining a stable interface. This process, which is specific rather than random, probably occurs due to intrinsic affinity (Figure 2). It is thus logical that increasing the folding rate of GFP decreases the extent of oligomerization. It has been reported that Superfolder GFP could escape cysteine-mediated oligomerization [23], and we also confirmed that SuperFast GFP formed less amounts of covalent dimers albeit it did not eliminate formation of the oxidized dimer (Figure 1, lane 16). Since it was impossible to identify the residues that are responsible for inter-subunit association during folding, we instead tested the possibility that cysteine residues could be substituted with other amino acids. Although the new set of fluorescent tags described here do not seem to hamper or slow the folding process, caution is needed since the self-assembling property of fluorescent proteins during folding may not be eliminated.

The surprising difference in folding and targeting of the prion fusion protein by the presence of cysteines in the tag indicates that folding of a tag *per se* could have global effects on the tagged target (Figures 6 and 7). The prion protein has been extensively studied as a cause of transmissible spongiform encephalopathies. Conformational alteration to the β-sheet rich structure is thought to be associated with the main pathogenic event [43]. Since it was beyond the scope of this study, we have not determined whether the SDS-resistant form of the prion fusion protein described in Figure 6 is related to the pathogenic form, which exhibits proteinase K resistance. This unusual property might have appeared because the SGFP2 tag containing an artificial signal sequence was fused to the N-terminus of prion at K23. Several studies have successfully demonstrated proper targeting of EGFP-prion fusion protein to the cell surface and generation of a transgenic mouse expressing the fusion protein when EGFP was inserted at the N-terminus of the GPI-attachment site [44] or at the C-terminus of the signal sequence cleavage site [45].

Since the thiol groups of two cysteine residues in GFP family members are located inside the tight β-can structure, it was not surprising that disulfide bonded oligomers emit no fluorescence. However, why then do the cysteine residues that are supposedly protected from solvent have an effect on diffusion? One possibility is that the oligomers caused a crowding effect on the normal diffusion of the fluorescent monomer. We think this unlikely because 1) the expression levels of two proteins (SGFP2 and cfSGFP2) were comparable and 2) measurements were performed at the sub-nanomolar, single molecule time regime. The second possibility is that large oligomers may simply block the narrow lumen of the ER. Strictly speaking, we could not exclude this and, indeed, the analysis using a confined diffusion model (Figure S1B) may be interpreted that the freely diffusing space was reduced in SGFP2. However, we think it unlikely because expression of the GFP-mCherry tandem dimer showed no punctate structures (our unpublished observation). Another possibility we think most likely is that fluctuation of a β-can structure may externalize cysteine residues so that they may react with other thiols or thiol-reacting enzymes such as polypeptide disulfide isomerase (ER) or thioredoxin (cytoplasm). This could happen stochastically, which would explain the time evolution of their diffusion. It should be noted that this cysteine-induced abnormality in motion is clearly irrelevant to the latrunculin-sensitive effects of N-linked oligosaccharides as shown previously [32] although the motion dynamics of a single molecule in the ER was studied by using conventional EYFP carrying two cysteine residues. We have confirmed that addition of two N-glycans to ss-cfSGFP2 caused severe distortion of simple diffusion, which disappeared by the addition of latrunculin B (our unpublished observation).

In this present study, we were able to find all of the functional alternative amino acids of cysteine in SGFP2, TagRFP and mKate2. Photochemical properties were largely unchanged by the mutations except a small blue-shift of excitation maxima (Table 1). A tendency of higher in vivo brightness of SGFP2 compared to lower extinction coefficient was also conserved in cfSGFP2. Conventionally, cysteine is replaced with serine because of its chemical similarity. However, none of the serine substitutions were successful to replace cysteine in the cylindrical β-can structure of the tags. Interestingly, all of the successful substitutions were aliphatic amino acids. In most cases, we identified only one amino acid that did not impair brightness. This observation may be related to the role of cysteine in the transmembrane helix bundle [46]. Cysteine is often found in the membrane-spanning domain of proteins. In phospholamban, a 52-residue integral membrane protein that forms a pentameric structure, three cysteine residues are located in the membrane spanning region and do not form disulfide bonds but their replacement with serine, alanine or phenylalanine largely affects the oligomeric stability of the protein. Reconstitution of chemically synthesized phospholamban showed that those cysteine residues could be structurally and functionally replaced with α-amino-*n*-butyric acid, which is isosteric to cysteine. This study suggests that cysteine could play a steric packing, but not nucleophilic role in a certain environment. Cysteine of FPs is likely to play a similar role in the seamless structure of FP β-can.

## Materials and Methods

### Plasmid Construction

SGFP2 was generated as described [24] by mutating EGFP-N1 (Invitrogen). cfSGFP2 was generated by introducing C48S first, then performing site-saturated mutagenesis on C70 using a primer as described in the Table S1. All PCR reactions were performed using KOD-plus DNA polymerase (TOYOBO, Tokyo) and mutagenesis was done according to a method as described in [47] or using a QuikChange Multi Site-Directed Mutagenesis kit (Stratagene). “Superfast” was generated as described [26]. The mutation A206K which reduces inter-subunit association [25] was introduced in EYFP (Invitrogen). Cys-containing mCherry was made by mutating a serine residue (S3C), which is outside the β-can structure, by using the primer listed in the Table S1. Similarly, the cysteine residues of TagRFP (Evrogen) were replaced with alternative amino acids. First, C222 was mutated to serine using a primer listed in the Table S1, and three consecutive rounds of site-saturated mutagenesis was performed on C26, C118 and C172 using the primers as listed in the Table S1, was further modified to remove the Asn-glycosylation site, N71, using the primer listed in the Table S1. To obtain a red-shift variant of cgfTagRFP, we initially applied the same set of mutations as cgfTagRFP, however, the C172V mutation in mKate2 failed to emit fluorescence. By screening another round of saturated mutagenesis on C172 using the primer listed in the Table S1, we identified C172A which retained the fluorescent property of mKate2 and this was called cgfmKate2.

For expression in the ER, the cleaved signal sequence of α1-antitrypsin (M1-K34) with appropriate restriction sites as listed in the Table S1 was fused to the N-terminus of SGFP2, cfSGFP2 or mCherry at BglII and EcoRI sites. In this case, the fragment M1-A24 should be removed cotranslationally after translocation. The long flanking sequence E25-K34 of α1-antitrypsin was included to ensure access to signal peptidase.

For bacterial expressions, a BamHI-NotI fragment encoding EGFP was cloned into the pGEX-6P vector (GE HealthCare, Tokyo). The cDNA fragments of SGFP2, cfSGFP2, TagRFP, mKate2, cgfTagRFP and cgfmKate2 were cloned into BamHI-HindIII sites of pQE-30.

The cDNA of the mouse prion protein was isolated by reverse-transcription of mRNA from NIH 3T3 cells and an entire coding region from K23, which is the N-terminus after signal sequence cleavage, to the stop codon was amplified by PCR using a set of primers as shown in the Table S1. The fragment was ligated into the BsrGI-MfeI sites of ss-SGFP2 or ss-cfSGFP2. The open reading frames of all plasmid constructs were sequenced and confirmed.

### Biochemical Analysis

The expression plasmids described above were transfected into COS7 cells cultured on 4 well dishes using polyethylenimine (Aldrich, code 408727) [48]. Brefeldin A (5 µm/ml) was added to prevent exit from the ER at 12 hr post-transfection. After 4 hr, cells were treated with 10 mM iodoacetamide (IA) for 10 min at room temperature to prevent artificial disulfide-bond formation in vitro, and then lysed with 1 mM IA/1% SDS/1 mM EDTA/10 mM Tris-Cl, pH8.8 [49]. DTT was added to half of each lysate at 10 mM to ensure complete reduction of the proteins. SDS denaturation was performed at either 70°C or 95°C for 10 min as described in the figure legends. The samples were subjected to SDS-PAGE using 9% acrylamide gels followed by immunoblotting with anti-calnexin, anti-GFP, anti-mRFP. or anti-TagRFP antibody essentially as described [50]. Rabbit antisera against EGFP, DsRed or TagRFP were generous gifts from Drs. Kenji Akasaki and Hiroshi Tsuji (Fukuyama University).

### Fluorescence Analysis of Living Cells

#### FCS analysis

Measurements of autocorrelation function in living cells were performed as described in detail previously [32]. Briefly, fluorescent proteins were expressed by loading expression plasmids with siliconized micro-glassbeads in 1% fetal bovine serum containing Minimum Essential Medium (MEM) that depleted phenolred/riboflavin/pyridoxal hydrochloride. To increase autocorrelation amplitudes, only the cells of least brightness, which were selected by visual inspection of fluorescence through a longpass filter at 505 nm, were used for measurements. Photon counting was carried out using ConfoCor2 (Carl-Zeiss) at 90 min-150 min post-plasmid-loading. Brefeldin A was included at 5 µm/ml throughout the experiments. To reduce interconversion between the bright state and dark state, a minimum laser output (0.29 µW of the 488 nm line from an argon-ion laser at the top of a 40x water-immersion objective) was used. The emission signals were detected through a >505 nm long-pass filter by the green channel for 488 nm excitation. To minimize distortion of the confocal volume in cells, measurements were done in a z-position that gave the highest count rates. The single component autocorrelation function is,
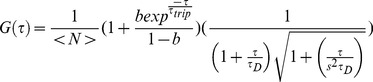
(3)where *b* and *τ_trip_* are the fraction and relaxation time of the triplet state, respectively, N is the mean molecule number in the confocal volume, *s* and *τ_D_* are the structural parameter and diffusional relaxation time. The equation for the multi-component analysis was descrbed previously [32]. The best fit numbers were obtained with Levenberg-Marquardt algorithm using OriginPro version 8.0 (OriginLab Corp).

To estimate the time dependency of diffusion coefficient, autocorrelation function including the anomalous exponent *α*,
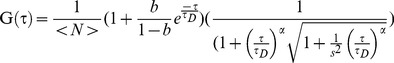
(4)was used. Autocorrelation function measurements were normalized and the averaged decay profiles were fitted with (4) (Figure S1).

Alternatively, if ER cargo motion is confined in a smaller space than the optical confocal volume, it may be appropriate to consider a model of diffusion limited by boundaries [31]. In this model, a free diffusing 3D autocorrelation model ignoring the triplet state population,
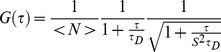
(5)is written as a product of three autocorrelation function *g_i_(τ)*,



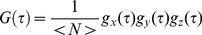
(6)We consider *r_z_* and *r_xy_,* distances in axial and lateral directions at which the intensity of the laser beam is dropped by *e* ^−2^, and *d_i_,* distance in *i*-direction at which boundary planes localize. The degree of confinement is expressed as *d_i_/r_i_*. If diffusion is confined in the optical axis and in x- and y-directions, *g_x_(τ)* is given as.
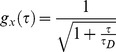
(7)


A closed form expression of *g_i_(τ)* is unknown, however, if *d_i_/r_i_≤*8, *g_i_(τ)* can be approximated as.
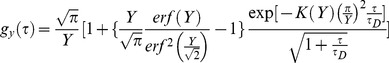
(8)

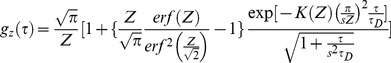
(9)where Y and Z are *d_y_/r_xy_* and *d_Z_/r_xy_,* respectively, and *K*(*i*) = 0.689+0.34*exp*(−0.37(*i*−0.5)^2^) [31]. Taking the triplet fraction into consideration, the FCS model becomes



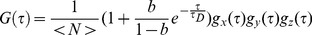
(10)Since estimation of the best-fit numbers requires calculation of error functions, we used FFS Data Processor ver. 2.3 (SSTC, Belarus).

#### PCH analysis

An observation volume of PCH is characterized by the point-spread function (PSF) of the instrument. For convenience, a scaled PSF, 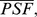 was considered, such that the volume defined for FCS (fluorescence correlation spectroscopy), is equal to the volume of 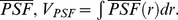 According to this model, the probability (*p^(1)^(κ;V_0_,ε)*) of detecting *k* photons from a single fluorescent molecule in a volume *V_0_* large enough to contain the illuminated volume is,
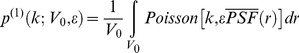
(11)where *ε* is the molecular brightness, which is a product of the average number of photon counts *<κ>* and a ratio *V_0_/V_PSF_*. For *n* independent molecules, total PCH is given by a convolution of the single molecule PCH. A 3D Gaussian approximation was originally proposed to describe the observation volume profile for one-photon excitation. However, different from FCS, an actual brightness profile by PCH is often disturbed by several optical factors such as photons in the out-of-focus region [51], [52]. Also, since the original assumption of PCH was that fluorescence intensity emitted by a molecule is constant, there is a clear effect of bin time for diffusing molecules [53]. Hence, we used a global analysis procedure that simultaneously obtains time-dependent decay of correlation function and PCH to recover relevant and common parameters [28].

Information on fluorescence intensity fluctuation was acquired using a confocal laser scanning microscope A1R (NIKON) equipped with a time-correlated single photon counting module (LSM Upgrade Kit, Picoquant). Expressions of fluorescent proteins were carried out in COS7 cells plated on 4-well glass bottom dish (Matsunami Glass Ind., Osaka, Japan) kept in a closed stage chamber (95% air and 5% CO_2_; Tokai Hit CO., Fujinomiya, Japan). The cells were excited using a 488 nm diode laser (85BCD020, Mellies Griot) or a 561 nm diode laser (85YCA010, Mellies Griot). The applied irradiance at the top of an objective lens was 5.3 µW (488 nm laser line) or 1.1 µW (561 nm laser line). The light signal was collected through a water-immersion objective (x60/NA1.27, Nikon) and split into two signals by a half-beam splitter. The two signals were fed to two separate single photon counting avalanche photodiodes (PDM Seires, MPD) through a bandpass filter (BP520/35, Semrock) for 488 nm excitation or a longpass filter (BA590, Nikon) for 561 nm excitation. The cross-correlation function of the two signals detected in the two photodiodes through the identical filter sets was used for accurate estimation of dynamic processes. The molecular brightness of each fluorescent protein was determined by finding a best-fit from globally linked datasets of a cross-correlation function and four PCH that were generated with bin time of 0.03, 0.06, 0.12 and 0.24 ms from one measuremt by using FFS data processor. A first-order correction for out-of-focus emission [51] and corrections for triplet and 3D diffusion were applied in the fitting as described [28].

### Single Molecule Imaging

Direct visualization of a single molecule in the ER was performed as described previously [32]. In Figure 4, COS7 cells were transfected with ss-SGFP2 expression vector using polyethylenimine and treated with brefeldin A for 4 hr as described above. They were then fixed in 4% paraformaldehyde/250 mM Hepes-Na, pH7.4 for 4 days at 4°C. The molecules in the ER of the cells were illuminated by evanescent light. A kymograph of each fluorescent spot was generated and the signals were quantified by ImageJ (ver 1.44).

### Measurement of Photo-chemical Properties

#### Purification of bacterially expressed proteins

Fluorescent proteins fused to the N-terminus of GST (glutathione-S-transferase) or polyhistidine tag were expressed in E coli XLI Blue (Invitrogen) and purified essentially as described [54]. The cells were grown at 37°C in LB medium (Becton Dickinson) containing ampicillin (100 µg/mL) overnight and diluted 50-fold with the LB medium. Protein expression was induced by the addition of IPTG (0.2 mM) when OD_600_ reached 0.4 and the cells were further incubated at 25°C for 14 hr. The cells collected by centrifugation were once frozen and resuspended in the ST buffer (150 mM NaCl/10 mM Tris-Cl, pH 8.0) containing 0.2% Triton X-100 and 1 mM phenylmethylsulfonyl fluoride, and then sonicated in an ice bath with Vibra-Cell (VCX600, Sonics & Materials). The homogenates were cleared by centrifugation at 10,000×g for 20 min and the supernatant was filtered through a cellulose acetate filter with a pore size of 0.8 µm (DISMIC-25cs, Millipore). The lysates were then applied onto a 1 mL column of gluthathione Sepharose 4B (GE Healthcare) for EGFP or a 1-mL Talon resin (TakaraBio, Ohtsu, Japan) for other fluorescent proteins. The unbound molecules were washed off with ST buffer containing 0.2% Triton X-100 and 10 mM imidazole and rinsed with ST. EGFP was cleaved off from the glutathione beads with PreScission Protease (GE Healthcare) according to the manufacturer’s protocol. All the other fluorescent proteins were eluted from Talon resin with 200 mM imidazole/150 mM NaCl/100 mM Hepes-OH, pH7.5). The proteins were further purified by a Mono Q column (GE Healthcare) chromatography and dialyzed against 20 mM Tris-Cl, pH 7.5.

#### Fluorescence quantum yield, extinction coefficient and spectra

The quantum yields of two independent preparations of the purified fluorescent proteins were measured with an absolute photoluminescence quantum yield measurement system (C9920-02, Hamamatsu Photonics, Japan), which is a fluorescence spectrophotometer equipped with an integrating sphere for compensating the effects of polarization and refractive index. The effects of reabsorption and reemission were corrected according to the manufacturer’s protocol as described in details by Suzuki et al [55]. Fluorescence spectra were also obtained by the measurements. An absorption spectrum was obtained by DU-800 (Beckman Coulter) and the molar extinction coefficient was determined by dividing the optical density with maximum absorbance by the molar concentration of the predicted molecular weights as described [22].

#### pKa measurements

Since formation of fluorophore can be monitored by measuring absorbance at the excitation maximum, we monitored their changes upon pH shift to estimate pKa values. Aliquotes (0.5 µL) of the fluorescent protein samples were diluted with 1.5 µL of 67 mM of titration buffers; citric acid-sodium citrate (pH 2.5–5.5), potassium phosphate monobasic-sodium phosphate dibasic (pH 6–8), or glycine-sodium hydroxide (pH 8.5–10) and the absorption spectrum were measured with NanoDrop2000c (ThermoScientific). The absorbances at the excitation maxima at each pH were plotted and pKa was determined by fitting the data to the Henderson-Hasselbalch equation with Levenberg-Marquardt algorithm using OriginPro version 8.0 (OriginLab Corp).

#### Photobleaching kinetics

COS7 cells cultured in a coverglass dish (MatTek) were transfected with an expression plasmid encoding each fluorescence protein as above and cultured on an inverted microscope (ECLIPSE-Ti-E, Nicon) at 32°C. The cells were then illuminated with the intense light (0.64 mW at the top of a 60x water-immersion objective (numerical aperture 1.27) from a 488 nm diode laser (Sapphire 488-200, Coherant) or a 561 nm diode laser (Jive 100, Cobolt) using a “full illumination” mode of N-SIM (NIKON). In this mode, non-structured lights illuminated the samples. The laser intensity was measured using a laser power meter, LP1 (SANWA, Japan). The images were recorded with an EM-CCD camera, iXon+ (DU-897, Andor) and fluorescence from an entire cell image were measured with NIS-Elements (ver 4.0.00, Nikon). Time constants τ of the decay were determined by curve fitting a single exponential decay to the mean fluorescence over time of six cells.

### Other Methods

For immunostaining, COS7 cells were fixed in 4% paraformaldehyde/250 mM Hepes-Na, pH7.4 for 1 hr on ice and immunolabeled with antigen affinity-purified antibody in 10% BlockAce (Dainippon Pharmaceutical Co, Osaka, Japan) containing “IF buffer” (0.15 M NaCl/2 mM MgCl_2_/1 mM EGTA/10 mM PIPES-Na, pH7.6) for 20 min after permeabilization on ice for 30 sec with 0.05% saponin in IF buffer. The antibody signal was visualized by incubating with anti-rabbit antibody conjugated to Alexa488 or Alexa555 (Invitrogen) for 20 min on ice. Confocal images of the fluorescence were taken by A1R confocal microscopy (Nikon) with a 60x oil-immersion objective (numerical aperture 1.49).

## Supporting Information

Figure S1 **Fitting of autocorrelation function of ss-cfSGFP2 or ss-SGFP2 with an anomalous model and a confined 3D diffusion model.** Decay profile of ss-cfSGFP2 or ss-SGFP2 (Figure 5) was fitted to an anomalous subdiffusion model or a confined 3D diffusion model as described in Methods. (A) Best fit and residuals for diffusion time *τ_D_* and anomalous factor *α* were obtained for the anomalous diffusion equation described in Materials and Methods. (B) Fitting to a confined 3D diffusion model. For simplicity, *d_z_/r_z_* and *τ_D_* were shared and only the best-fit values of *d_y_/r_xy_* were determined for each protein. The best fit *d_z_/r_z_* was 1.6. The fits (red line) and residuals were shown.
(TIF)

Figure S2 **Effects of cysteine on the free diffusion of the cytoplasmic protein**. Autocorrelation function amplitudes of SGFP (black line, n = 151) or cfSGFP2 (red line, n = 193) in the cytoplasm of living cells were measured and normalized. The average decay profiles of each measurement is shown in panel A as a mean (left) or median (right) and the median (*cross*) and CI95 (perpendicular bold line) in a range of τ = 0.04–12 ms are shown in panel B. Variance of the SGFP2 autocorrelation function of cfSGFP2 was observed in the diffusion time regime. Each median correlation curve was globally fitted to a two component diffusion model. The best-fit values are shown in (C) and residuals in (D).
(TIF)

Figure S3 **pKa determination of newly developed fluorescent probes**. Loss of excitation absorbance at 493 nm (cfSGFP2), 555 nm (cgfTagRFP) or 584 nm (cgfmKate2) was plotted. The best-fits to Henderson-Hasselbalch equation were determined as shown in Table.
(TIF)

Video S1 **Live cell imaging of ss-cfSGFP2-prion fusion protein.** COS7 cells transiently expressing ss-cfSGFP2-prion was exposed to 0.5 mM CuSO_4_ in phosphate-buffered saline at pH 5.0 for 3 min at room temperature and images were continuously recorded at 32°C at 1 frame per min. The z-position of images from an objective lens was set constant using the PerfectFocus function of A1R confocal microscopy. Apparent apoptotic cell death after internalization of large aggregates was observed in some cells (*left end*). The width and height of images were 81.9 µm.
(MP4)

Table S1 (DOC)
