# Supplementary figures and images for: Development of Cysteine-Free Fluorescent Proteins for the Oxidative Environment
Source: PLoS One. 2012 May 23;7(5):e37551. doi: 10.1371/journal.pone.0037551 (PMC3359384; doi:10.1371/journal.pone.0037551)

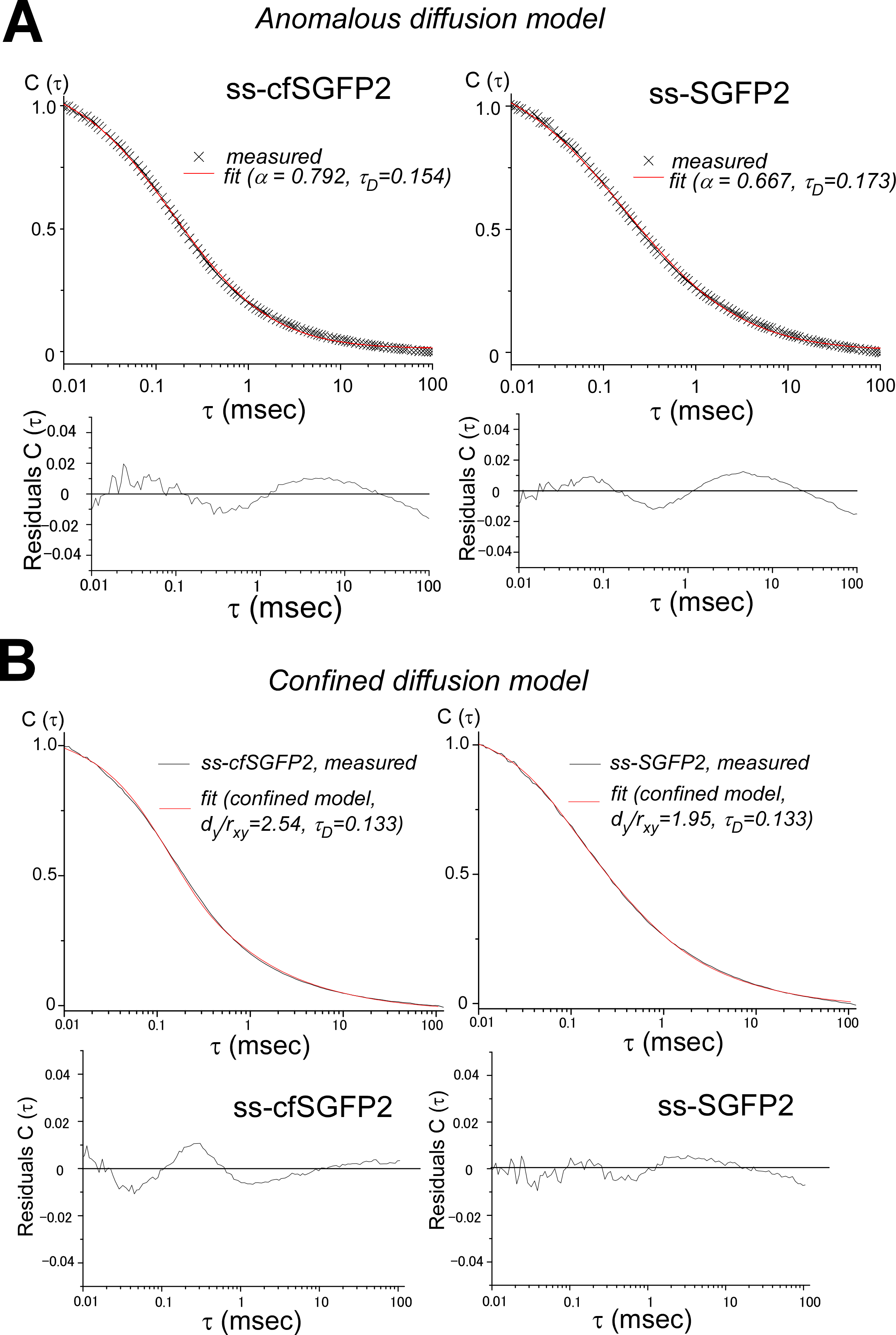

Supplement: Figure S1 — Fitting of autocorrelation function of ss-cfSGFP2 or ss-SGFP2 with an anomalous model and a confined 3D diffusion model. Decay profile of ss-cfSGFP2 or ss-SGFP2 (Figure 5) was fitted to an anomalous subdiffusion model or a confined 3D diffusion model as described in Methods. (A) Best fit and residuals for diffusion time τD and anomalous factor α were obtained for the anomalous diffusion equation described in Materials and Methods. (B) Fitting to a confined 3D diffusion model. For simplicity, dz/rz and τD were shared and only the best-fit values of dy/rxy were determined for each protein. The best fit dz/rz was 1.6. The fits (red line) and residuals were shown. (TIF) [file pone.0037551.s001.tif]

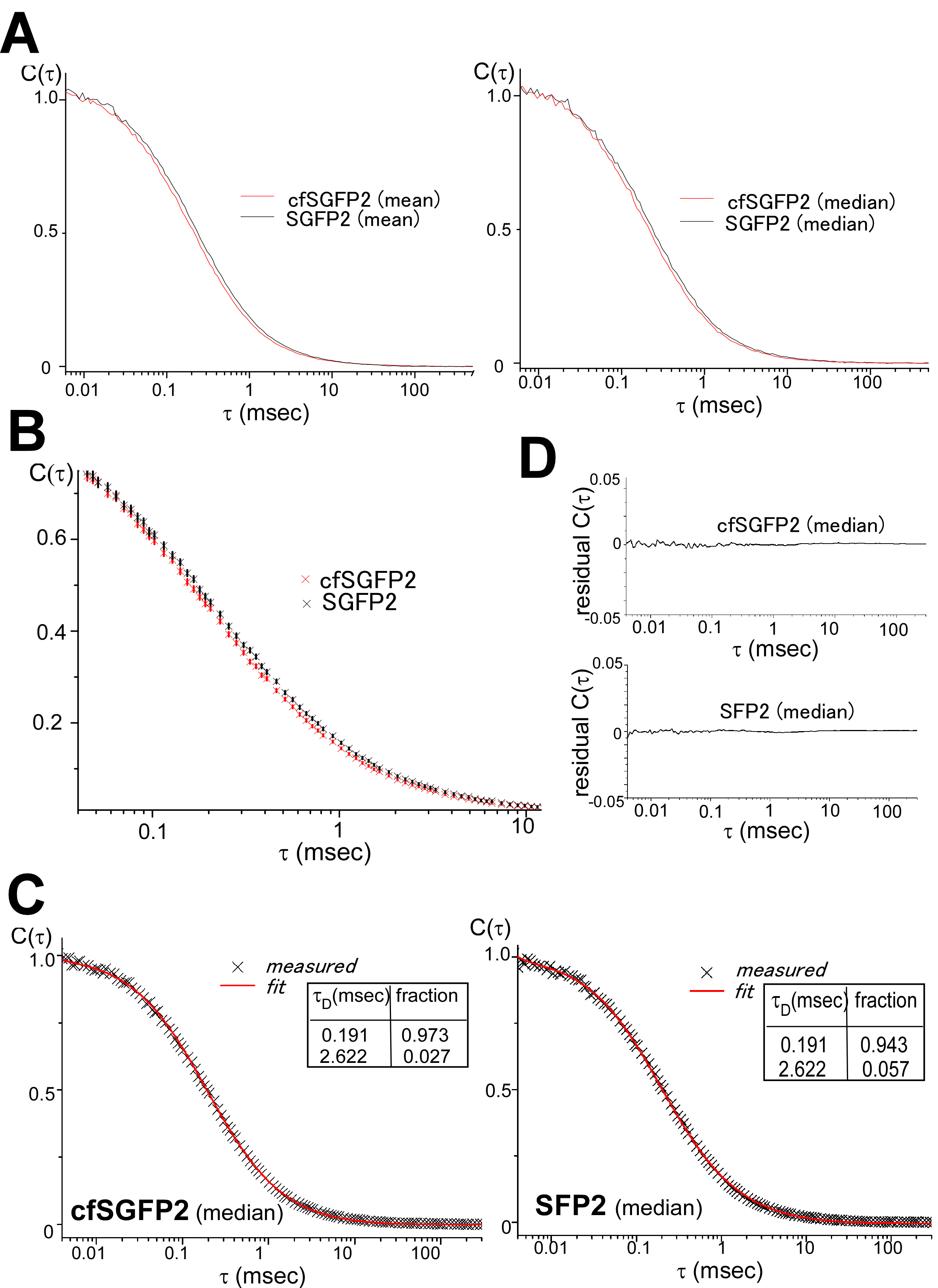

Supplement: Figure S2 — Effects of cysteine on the free diffusion of the cytoplasmic protein. Autocorrelation function amplitudes of SGFP (black line, n = 151) or cfSGFP2 (red line, n = 193) in the cytoplasm of living cells were measured and normalized. The average decay profiles of each measurement is shown in panel A as a mean (left) or median (right) and the median (cross) and CI95 (perpendicular bold line) in a range of τ = 0.04–12 ms are shown in panel B. Variance of the SGFP2 autocorrelation function of cfSGFP2 was observed in the diffusion time regime. Each median correlation curve was globally fitted to a two component diffusion model. The best-fit values are shown in (C) and residuals in (D). (TIF) [file pone.0037551.s002.tif]

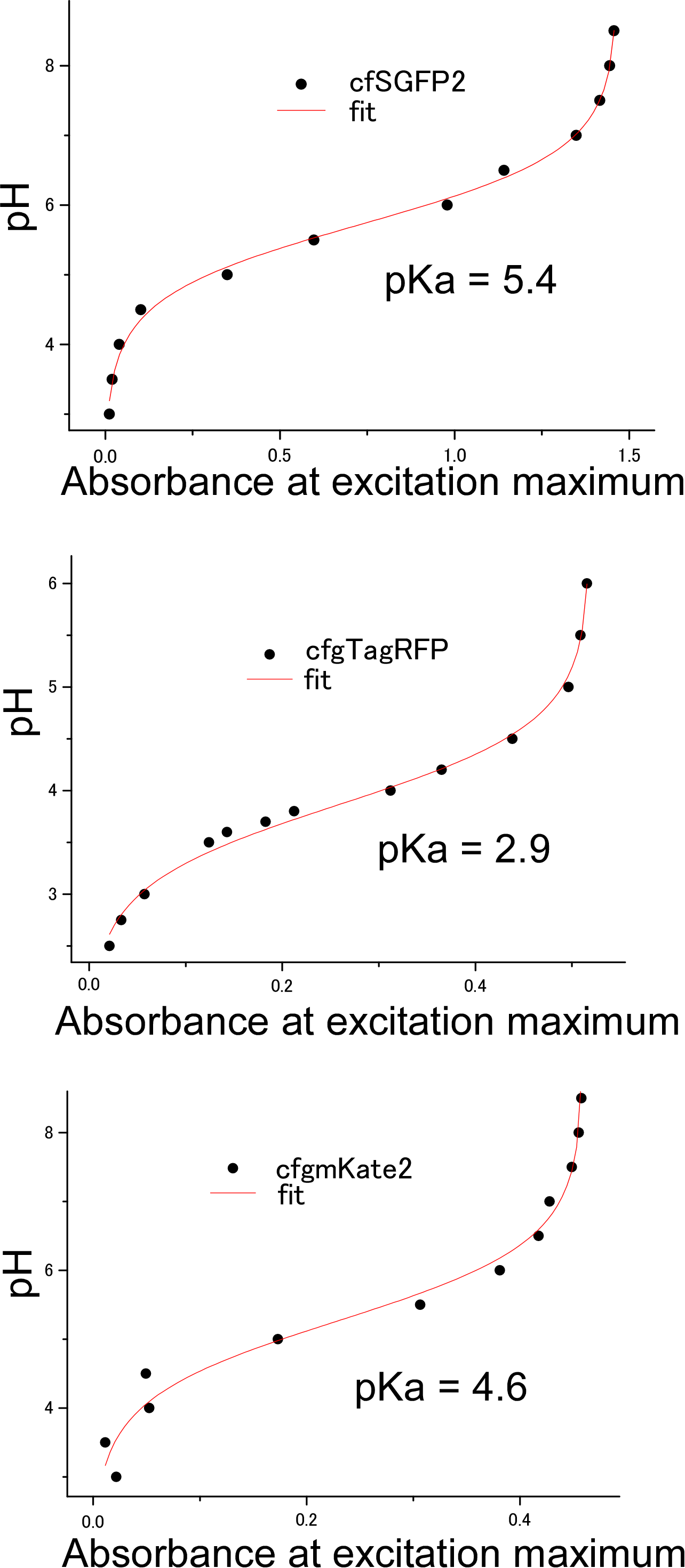

Supplement: Figure S3 — pKa determination of newly developed fluorescent probes. Loss of excitation absorbance at 493 nm (cfSGFP2), 555 nm (cgfTagRFP) or 584 nm (cgfmKate2) was plotted. The best-fits to Henderson-Hasselbalch equation were determined as shown in Table. (TIF) [file pone.0037551.s003.tif]
