# Supplementary material for: Development of Cysteine-Free Fluorescent Proteins for the Oxidative Environment
Source: PLoS One. 2012 May 23;7(5):e37551. doi: 10.1371/journal.pone.0037551 (PMC3359384; doi:10.1371/journal.pone.0037551)
Supplement: Table S1 — (DOC) [file pone.0037551.s005.doc]

Supplemental Table

Nucleotides that are different from the template are in bold face.

| Template | Mutations/ restriction site | Oligonucleotide sequence (5’ – 3’) |
| --- | --- | --- |
| SGFP2, PA-SGFP2 | C48S | ACCCTGAAGTTCATCT**C**CACCACCGGCAAGC |
| EYFP | A206K | CTGAGCTACCAGTCC**AAA**CTGAGCAAAGACCC |
| SGFP2(C48S)  PA-SGFP2(C48S) | C70 | CTGACCTACGGCGTGCAG**NNN**TTCAGCCGCTACCCCG |
| mCherry | S3C | CCACCATGGTGTGCAAGGGCGAGG |
| tagRFP(C222S) | C26 | CAACCACCACTTCAAG**NNN**ACATCCGAGGGC |
| tagRFP(C26A) | C118 | CTCCAGGACGGC**NNN**CTCATCTACAACG |
| tagRFP(C26A /C118M), mKate2 | C172 | GGGCCACCTGATC**NNN**AACTTCAAGAC |
| tagRFP | C222S | CTGTGGCCAGATACT**CG**GACCTCCCTAG |
| eba2 | N71 | GCAGCAGAACCTTCATC**NNN**CACACCCAGGGCATCCC |
| Antitrypsin  (human) | BglII | GT**AG**ATC**T**ACAATGCCGTCTTCTGTCTCGTGG |
| Antitrypsin  (human) | EcoRI | TG**G**A**AT**T**C**TCTTCTGGGCAGCATCTCCCTG |
| Prion mRNA |  | CCAGGGCCCATCAGTGCCAG |
| Prion cDNA | BsrGI | ATGTCGGCCT**G**T**A**CAAAAAGCGGCCAAAGC |
| Prion cDNA |  | GCCTATGGGGGACACAGAGAAGCAAGAATG |
